# BCG Vaccination Suppresses Glucose Intolerance Progression in High-Fat-Diet-Fed C57BL/6 Mice

**DOI:** 10.3390/medicina60060866

**Published:** 2024-05-25

**Authors:** Haruna Arakawa, Masashi Inafuku

**Affiliations:** 1 Faculty of Agriculture, University of the Ryukyus, Senbaru 1, Nishihara 903-0213, Japan; hana20001714@yahoo.co.jp; 2 The United Graduate School of Agricultural Sciences, Kagoshima University, Kagoshima 890-0065, Japan

**Keywords:** BCG, glucose intolerance, insulin resistance, trained immunity, nonalcoholic fatty liver disease

## Abstract

*Background and Objectives*: *Mycobacterium bovis* Bacillus Calmette–Guérin (BCG) vaccine administration has been suggested to prevent glucose metabolism abnormalities and fatty liver in genetically obese *ob/ob* mice; however, it is not clear whether the beneficial effects of BCG are also observed in the progression of glucose intolerance induced by a high-fat diet (HFD). Therefore, the effects of BCG vaccination on changes in glucose tolerance and insulin response were investigated in HFD-fed C57BL/6 mice. *Materials and Methods*: We used the BCG Tokyo 172 strain to determine effects on abnormalities in glucose metabolism. For vaccination, five-week-old male mice were injected intraperitoneally with BCG and maintained on a HFD for three weeks. The mice were regularly subjected to intraperitoneal glucose tolerance and insulin tolerance tests (IGTTs and ITTs). These tests were also performed in mice transplanted with bone marrow cells from BCG-vaccinated donor mice. *Results*: Significant effects of BCG vaccination on blood glucose levels in the IGTTs and ITTs were observed from week 12 of the experiment. BCG vaccination significantly improved changes in fasting glucose and insulin levels, insulin resistance indexes, and glucagon-to-insulin ratios in conjunction with the HFD at the end of the experiment. Significant inhibitory effects in the IGTTs and ITTs on glucose intolerance were also observed with transplantation with bone marrow cells derived from BCG-vaccinated donor mice. *Conclusions*: BCG vaccination significantly delayed glucose intolerance progression, suggesting a beneficial effect of BCG on the pathogenesis of type 2 diabetes. It has also been suggested that the effects of BCG vaccination may be at least partially due to an immune memory (trained immunity) for hematopoietic stem and progenitor cells of the bone marrow.

## 1. Introduction

Obesity, especially visceral obesity, contributes to the pathogenesis of metabolic syndrome, a cluster of metabolic abnormalities including hyperlipidemia, hypertension, and insulin resistance (IR) [1]. It is well known that IR is the primary indicator of type 2 diabetes mellitus (T2DM). T2DM pathogenesis is also considered to be linked to the innate and adaptive immune systems, which are recognized as important etiological components in the development of IR [2,3,4]. Per the International Diabetes Federation, T2DM is the most common type of diabetes (accounting for approximately 90% of all cases). In 2021, more than one in ten adults had diabetes mellitus globally, and the number of people with this disease will continually increase in the future [5]. The increasing prevalence of this condition makes it a public health problem of paramount importance. T2DM imposes a significant personal and public health burden in terms of the number of people affected, complications, and expenses incurred by national health and social care systems [6]. Therefore, the discovery and development of new treatments that regulate glucose and metabolic homeostasis are urgently needed.

An imbalance in energy homeostasis is a hallmark of T2DM [7]. Altered immune surveillance and impaired host defenses have been observed in patients suffering from obesity and T2DM, which may predispose patients to infection caused by germs such a *Mycobacterium tuberculosis* (Mtb) [8,9]. Animal and human studies have also indicated an increased susceptibility to Mtb infection in type 1 diabetes mellitus (T1DM), which is commonly known as juvenile-onset diabetes and characterized by an absolute deficiency in insulin production by the autoimmune destruction of islet β-cells [10,11]. To protect against Mtb infection and its progression to tuberculosis, an attenuated strain of *M. bovis* was used to develop the Bacillus Calmette–Guérin (BCG) vaccine over 100 years ago. The nonspecific effects of BCG were first used for bladder cancer treatment over 40 years ago [12]. Thereafter, the off-target effects of BCG have been shown to protect against infectious and noninfectious diseases, including T1DM [13,14,15,16,17,18]. It has been reported that repeated BCG vaccinations in long-term diabetics can restore blood sugars to near normal by resetting the immune system and by increasing glucose utilization through a metabolic shift to aerobic glycolysis, a high-glucose-utilization state. [18]. However, to the best of our knowledge, only a few studies have examined the effects of BCG vaccination on T2DM. It has also been reported that in leptin receptor-deficient *db/db* mice, multiple BCG injections significantly decreased blood glucose levels and increased glucose uptake in bone marrow cells [19]. Our previous study demonstrated that a single intravenous administration of BCG significantly decreased serum insulin levels and the insulin resistance index in a homeostatic model assessment for insulin resistance (HOMA-IR) in leptin-deficient *ob/ob* mice [20]. However, to the best of our knowledge, no information is available on the effect of BCG vaccination on diet-induced glucose intolerance. In the current study, we investigate the effects of prior BCG vaccination on the progression of glucose intolerance in high-fat and chow-diet-fed mice.

## 2. Materials and Methods

### 2.1. Animals, Diet, and Microorganisms

All animal experiments were approved by the Animal Care and Use Committee of the University of the Ryukyus (approval numbers: A2022003 and A2022007) and conducted per their guidelines. Male C57BL/6JmsSlc (CD45.2; referred to as B6-Ly5.2) mice were purchased from Japan SLC Inc. (Shizuoka, Japan). C57BL/6-Ly5.1 (CD45.1; referred to as B6-Ly5.1) mice were maintained in our animal laboratory. The mice were randomly housed in environmentally enriched cages (5 animals per cage) under a controlled environment (at 24 °C ± 1 °C in a 12 h day/night cycle with lights on from 07:00 to 19:00). After one week of adaptation, the mice were randomly divided into experimental groups (n = 10/group) for each experiment. All animals had free access to food and water during the experiment.

Products for a commercial chow diet (12 kcal% fat, CE-2 diet) and a high-fat diet (HFD, 30 kcal% fat, Quick fat diet) were purchased from CLEA Japan, Inc. (Tokyo, Japan). The BCG Tokyo 172 strain was purchased from the Japan BCG Laboratory (Tokyo, Japan) and suspended at 5 × 10^8^ colony-forming units (CFU)/mL in phosphate-buffered saline (PBS) before use. 

### 2.2. BCG Vaccination in HFD-Fed Mice

**Figure 1:**
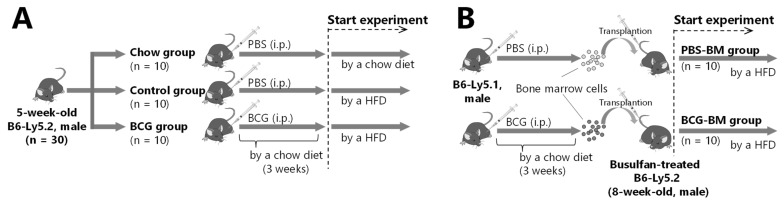
Schematic diagram of the experimental design. (**A**) To assess the effect of BCG vaccination on glucose intolerance progression in HFD-fed mice. (**B**) To assess the effect of bone marrow transplantation from BCG-vaccinated mice on glucose intolerance progression in HFD-fed mice. PBS-BM group were transplanted with bone marrow from PBS-treated mice; BCG-BM group were transplanted with bone marrow from BCG-vaccinated mice.

For vaccination, five-week-old-B6-Ly5.2 male mice were intraperitoneally (i.p.) injected with BCG (5 × 10^7^ CFU/100 µL) for the BCG group, and with vehicle PBS for the chow and control groups (Figure 1A). All mice were fed a chow diet for three weeks, after which the chow group continued on the chow diet, and the control and BCG groups transferred to a HFD for the rest of the experiment. After the thirty-week feeding period, the mice were sacrificed after 12 h of starvation by exsanguination from the heart under isoflurane anesthesia to minimize suffering. 

### 2.3. Bone Marrow Transplantation from BCG-Vaccinated Mice

Donor B6-Ly5.1 male mice were i.p. injected with vehicle PBS or BCG (5 × 10^7^ CFU/100 µL) and then fed a chow diet for three weeks (Figure 1B). Their bone marrow samples were obtained by flushing their femurs and tibias with Eagle’s minimum essential medium, followed by resuspension in PBS for transplantation. Recipient 8-week-old B6-Ly5.2 male mice were treated with i.p. busulfan injection for five days (20 mg/kg body weight/day) and transplanted intravenously with 1 × 10^7^ nucleated cells from the donor’s bone marrow 24 h after the last busulfan injection. In this experiment, mice transplanted with bone marrow cells derived from PBS-treated mice served as the PBS-BM group, and those from BCG-vaccinated mice served as the BCG-BM group. After bone marrow transplantation, all mice were fed an HFD.

### 2.4. Intraperitoneal Glucose Tolerance Test and Insulin Tolerance Test

At 8, 12, and 24 weeks after the start of the experiment, intraperitoneal glucose tolerance tests (IGTTs) were performed to assess whether the mice exhibited alterations in peripheral glucose regulation. The mice fasted for 12 h and were injected i.p. with D-glucose (1 g/kg body weight). Their blood glucose levels were measured before and at 30, 60, 90, and 120 min post glucose injection. For the insulin tolerance test (ITT), mice fasted for 4 h and were injected with human recombinant insulin (1 U/kg body weight; Wako Pure Chemical Industries, Ltd., Osaka, Japan) i.p., and blood glucose levels were measured before and at 30, 60, 90, and 120 min post injection. For both tests, blood was obtained from the tail vein, and glucose levels were measured using a glucometer (Free Style Precision Neo, Abbott Laboratories, Green Oaks, IL, USA).

### 2.5. Measurement of Biochemical Parameters in Serum

Serum triglyceride (TG), total cholesterol and glucose levels, and the activities of hepatopathy indicators, as well as the activities of alanine aminotransferase (ALT) and aspartate aminotransferase (AST), were measured using a commercial enzymatic kit (Wako Pure Chemical Industries, Ltd., Osaka, Japan). Serum insulin and glucagon levels were measured using enzyme-linked immunosorbent assay kits purchased from Morinaga Institute of Biological Science, Inc. (Kanagawa, Japan), and Wako Pure Chemical Industries, Ltd., respectively. The homeostatic indexes for the quantification of insulin resistance and beta cell function (HOMA-IR and HOMA-β) were calculated as previously described [21]. 

### 2.6. Measurement of Hepatic Lipid Levels

Hepatic lipids were extracted and purified using a previously reported method [22]. We determined hepatic TG levels using commercial enzymatic kits (Wako Pure Chemical Industries).

### 2.7. Histopathological Examination

The pancreas was excised and immediately fixed in 10% neutral formalin solution. Formalin-fixed samples were embedded in paraffin and cut into 4 µm thick sections. Paraffinized tissue sections were stained with hematoxylin and eosin (H&E) per a standard protocol for microscopic evaluation. The sizes of islets were calculated from digital images using Image J software (version 1.54i, NIH, Bethesda, MA, USA).

### 2.8. Flow Cytometry

The monoclonal antibodies (mAb) used in this study included APC/Cyanine7-conjugated anti-mouse CD45.1 mAb (clone A20), PerCP-cyanine5.5-conjugated anti-mouse CD45.1 mAb (clone 104), and non-labeled anti-mouse CD16/CD32 mAb (clone 2.4G2), bought from BioLegend Inc. (San Diego, CA, USA), Thermo Fisher Scientific Inc. (Waltham, MA, USA), and BD Biosciences (Milpitas, CA, USA), respectively. Before staining with the labeled mAb, isolated splenocyte was preincubated with anti-CD32/CD16 mAb (2.4G2, BD Biosciences) to prevent the nonspecific Fc-receptor-mediated binding of mAbs. The stained cells were analyzed on a FACSCanto II flow cytometer with the FACSDiva software program (version 5.0, BD Biosciences).

### 2.9. Statistical Analyses

All data are expressed as the mean ± SEM. The statistical significance of the difference between the two experimental groups was determined using the Student’s *t-*test. To determine the significance of the differences among mean values in the three experimental groups, the differences among the mean values were inspected using the Tukey–Kramer multiple comparison test. The threshold for statistical significance was set at *p* < 0.05.

## 3. Results

### 3.1. Effect of BCG Vaccination on Glucose Intolerance in HFD-Fed Mice

**Figure 2:**
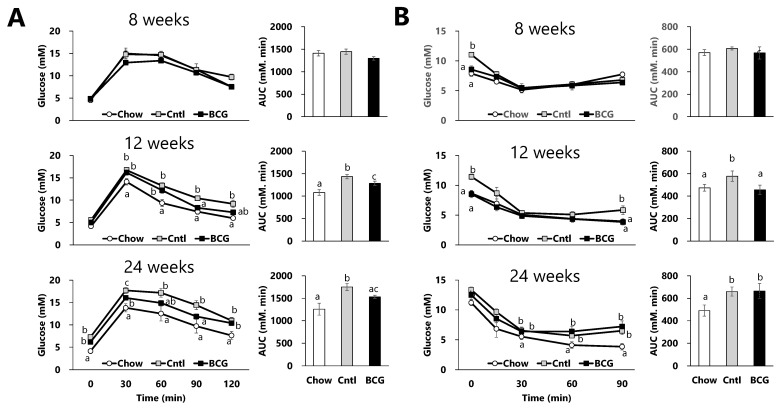
Effect of BCG vaccination on the progression of glucose intolerance in high-fat-diet-fed mice. (**A**) Effect of BCG vaccination on the intraperitoneal glucose tolerance test (IGTT). (**B**) Effect of BCG vaccination on the insulin tolerance test (ITT). Chow; chow group, Cntl; control group, BCG; BCG group. Data are shown as the mean ± SEM. Different letters indicate significant differences among the experimental groups using Tukey–Kramer multiple comparison test (*p* < 0.05).

To assess the effect of BCG vaccination on the progression of glucose dysmetabolism in HFD-fed mice, we performed IGTTs and ITTs to measure the ability of mice to retain circulatory glucose levels over time after administering glucose and insulin, and calculated the area under the curve (AUC) from these results. Changes in blood glucose levels and the AUC in the GTT at week 8 of the experimental period did not differ significantly among all experimental groups (Figure 2A). Although no significant differences were detected in fasting glucose levels, the blood glucose levels in the HFD-fed control group at all measurement time points after glucose administration and the AUC were significantly increased compared with those in the chow group at week 12. However, when comparing the mice in the HFD-fed groups, blood glucose levels at 90 and 120 min after glucose administration and the AUC in the BCG group were significantly lower than those in the control group. The IGTT performed at week 24 revealed that HFD feeding led to marked hyperglycemia; however, BCG vaccination significantly reduced these abnormal levels and suppressed the increase in the AUC. As shown in Figure 2B, blood glucose levels after 4 h of fasting in the chow and BCG groups were significantly lower than those in the control group, although there were no significant differences in the mean dietary intake among all experimental groups. Significant inhibitory effects of BCG vaccination on blood glucose levels after insulin administration were observed only at week 12 of the experimental period in this study.

### 3.2. Effect of BCG Vaccination on Growth Parameters, Blood Parameters, and Hepatic Lipid Content

We assessed the effects of BCG on growth and serum parameters (Table 1). HFD consumption resulted in significant increments in final body weight, liver weight, serum TG level, and hepatic TG content. BCG vaccination tended to inhibit these increments in liver weight and hepatic TG content. The serum cholesterol level and hepatopathy indicators did not differ significantly among all experimental groups. 

### 3.3. Effect of BCG Vaccination on Glucose Metabolism Parameters

**Figure 3:**
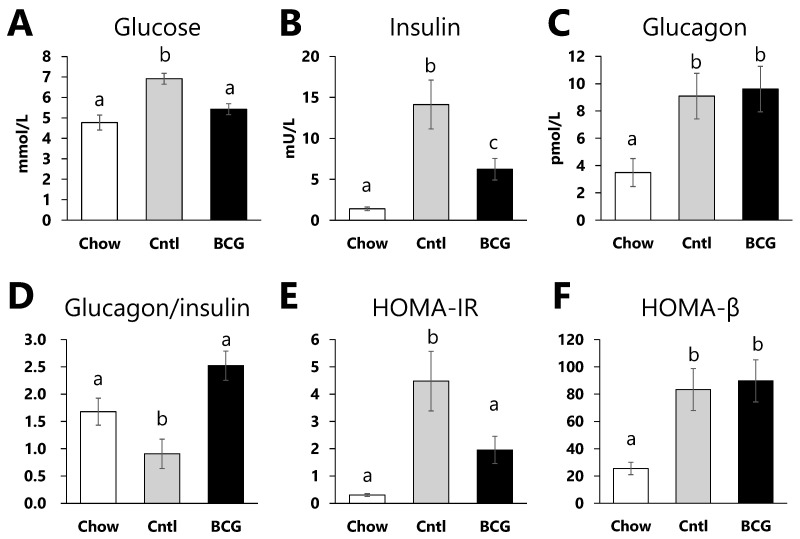
Effect of BCG vaccination on serum parameters related to glucose metabolism in high-fat-diet-fed mice at the end of the experiment. (**A**) Serum glucose level. (**B**) Serum insulin level. (**C**) Serum glucagon level. (**D**) Glucagon-to-insulin ratio. (**E**) Homeostatic model assessment for insulin resistance (HOMA-IR). (**F**) Homeostatic model assessment for beta cell function (HOMA-β). Chow: chow group; Cntl: control group; BCG, BCG group. Data are presented as the mean ± SEM. Different letters indicate significant differences among the experimental groups using Tukey–Kramer multiple comparison test (*p* < 0.05).

Fasting serum glucose, insulin, and glucagon levels in the control group were significantly higher than those in the chow group (Figure 3A–C). A significant decrease in the glucagon-to-insulin ratio and significant increments in HOMA-IR and HOMA-β in the control group were observed compared with those in the chow group (Figure 3D,E). BCG vaccination significantly decreased fasting serum glucose levels and insulin levels compared with the control group (Figure 3A,B). Although no significant effects of BCG vaccination on glucagon levels were observed (Figure 3C), the HFD-induced decrease in the glucagon-to-insulin ratio was significantly alleviated by BCG vaccination (Figure 3D). HOMA-IR in the BCG group was significantly decreased compared with that in the control group; meanwhile, HOMA-β did not differ significantly between the control and BCG groups (Figure 3E,F). 

### 3.4. Effect of BCG Vaccination on Pancreatic Islet Size

**Figure 4:**
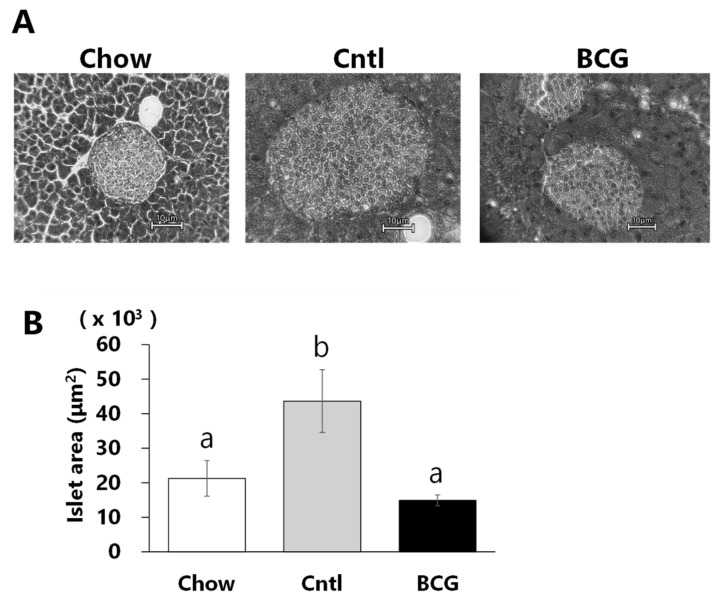
Effect of BCG vaccination on pancreatic islet size in high-fat-diet-fed mice at the end of the experiment. (**A**) Representative H&E histology of pancreatic samples from mice in each experimental group (scale bar = 100 μm). (**B**) The size of pancreatic islets. Chow: chow group; Cntl, control group; BCG, BCG group. Data are shown as the mean ± SEM. Different letters indicate significant differences among the experimental groups using Tukey–Kramer multiple comparison test (*p* < 0.05).

As shown in Figure 4, the sizes of the pancreatic islets in the control group were larger than in the chow group. These significant increments in islet size were inhibited by BCG-vaccinated mice.

### 3.5. Effect of Bone Marrow Transplantation from BCG-Vaccinated Mice on Glucose Intolerance Progression

**Figure 5:**
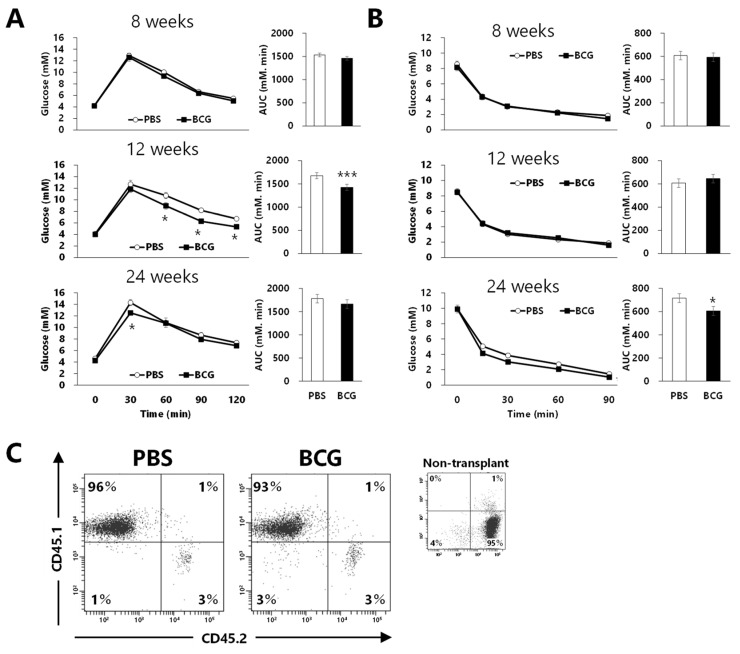
Effect of bone marrow transplantation from BCG-vaccinated mice on the progression of glucose intolerance in high-fat-diet-fed mice. (**A**) Effect of BCG vaccination on intraperitoneal glucose tolerance test (IGTT). (**B**) Effect of BCG vaccination on insulin tolerance test (ITT). (**C**) Representative flow cytometry plot of CD45.1 (Ly5.1) and CD45.2 (Ly5.2). PBS: PBS-BM group that were transplanted with bone marrow from PBS-treated mice; BCG: BCG-BM group that were transplanted with bone marrow from BCG-vaccinated mice. Data are shown as the mean ± SEM. The asterisk shows significant differences as compared with the PBS-BM group using the Student’s *t-*test (* *p* < 0.05, *** *p* < 0.001).

To assess whether immunomodulation induced by BCG vaccination affects the progression of glucose intolerance in HFD-fed mice, recipient B6-Ly5.2 mice were transplanted with bone marrow from donor B6-Ly5.1 mice in which BCG vaccination performed three weeks earlier had caused immune changes. At week 8, the results of the IGTTs and ITTs did not differ significantly between the PBS- and BCG-BM groups (Figure 5A,B). Blood glucose levels and AUCs were significantly decreased in the BCG-BM group compared with those in the PBS-BM group at week 12, although BCG vaccination did not affect the ITT results. Maximal blood glucose levels after 30 min of glucose administration were significantly decreased in the BCG-BM group compared with those in the PBS-BM group at week 24. Although blood glucose levels after insulin administration in the BCG-BM group tended to be lower than those in the PBS-BM group, the AUC of the BCG-BM group was significantly decreased compared to the PBS-BM group. More than 90% of the immune cells of recipient mice used in these studies were of donor origin (Figure 5C).

## 4. Discussion

BCG is a microorganism that was developed as a vaccine for tuberculosis 100 years ago, and its off-target effects have been found to range from cancer treatment to protection against infectious and noninfectious diseases [12,13,14,15,16,17,18]. Non-obese diabetic (NOD) mice are well-studied spontaneous models of autoimmune diabetes, but they mimic only some features of T1DM [17,23]. Three decades of research have indicated that BCG administration permanently cures diabetes when administered to NOD mice [23,24]. Many studies have shown that BCG has therapeutic promise for T1DM in humans [17,25,26,27]; meanwhile, others have suggested that BCG is not useful for T1DM [28,29,30]. T1DM onset is typically associated with the rapid loss of pancreas function from the T-cell autoimmune attack on the insulin-secreting cells of the islets of Langerhans. The impact of BCG on human blood sugars in T1DM appears to be driven by immune and immune–metabolic effects [31]. The transfer of immune cells from BCG-vaccinated NOD mice prevented the occurrence of overt diabetes in the recipients, while the transfer from untreated donors did not [23]. It has been demonstrated that BCG can reset the immune system on the cellular level by inducing suppressive regulatory T cells and killing the autoreactive cytotoxic T cells that attack insulin-secreting cells [32,33]. It is also known that aerobic glycolysis is suppressed in individuals with T1DM, and oxidative phosphorylation, which is a metabolic pathway involving low glucose utilization, high ketone production, and high Krebs cycle utilization, is predominant instead. Recent studies have shown that BCG treatment switches the systemic metabolism from overactivated oxidative phosphorylation to accelerated aerobic glycolysis, suggesting that this leads to the lowering of blood sugar levels [17,18]. This suggests that the BCG induction of aerobic glycolysis has broader applicability to other forms of hyperglycemia, including T2DM [17]. 

Our previous study suggested that intravenous BCG administration may be therapeutic in relation to the inhibition of fat accumulation and reducing fasting insulin levels and the insulin resistance index, HOMA-IR, in an obese T2DM model of leptin-deficient *ob/ob* mice [20]. Shpilsky et al. recently revealed a significant reduction in blood sugar levels and body weight gain by four BCG injections in the rear footpads of *db/db* mice in a model of T2DM, although a single injection failed to have a clinical outcome [19]. Herein, we examined the beneficial effects of single and prior BCG vaccination on HFD-induced glucose intolerance in wild-type C57BL/6 mice. Although significant effects of HFD intake on changes in blood sugar and the AUC in both the IGTTs and ITTs were observed from week 12 onward, blood glucose levels after 4 h of fasting were significantly lower in both the chow and BCG groups than in the control group at week 8 (Figure 2). These data suggest that prior BCG vaccination not only lowers blood glucose levels, but also inhibits glucose dysmetabolism progression in HFD-fed mice. 

At the end of the experimental period, fasting blood glucose, insulin levels, and HOMA-IR in BCG-vaccinated mice were significantly lower than in the control mice (Figure 3A,B,E). Fasting insulin levels and HOMA-IR are one approach to measuring IR [34]. T2DM is characterized by hyperglycemia, typically due to the interaction of IR and impaired beta cell function [35]. We observed that BCG vaccination does significantly not affect HOMA-β (Figure 3F). Pancreatic β-cell function is commonly estimated using HOMA-β, which in individuals with T2DM increases between years 4 and 3 before diagnosis, and then decreases until diagnosis [36]. Glucose intolerance in HFD-fed mice with reduced insulin sensitivity is suggested to impair β-cell function in pancreatic islets, resulting in excessive β-cell proliferation and increased islet size [37,38]. It has also been reported that the dietary intake of an HFD to induce IR in rodents results in an increase in pancreatic islet size [39,40]. The H&E staining showed that pancreatic islets were herein larger in the control group than in the chow group, and this increase was significantly diminished by BCG vaccination (Figure 4). These data suggest that prior BCG vaccination alleviates the development of β-cell dysfunction in HFD-fed mice and consequently improves insulin tolerance.

The glucagon-to-insulin ratio was ameliorated in the BCG group compared with that in the control group, although the serum glucagon levels were largely comparable between both experimental groups (Figure 3C,D). Recent studies have indicated that the lower the glucagon-to-insulin ratio, the greater the likelihood of suffering from nonalcoholic fatty liver disease (NAFLD), cardiovascular disease, and metabolic syndrome in patients with T2DM [41,42]. Intravenous BCG administration to *ob/ob* mice has been shown to improve the hepatic fat accumulation state and serum levels of high-molecular-weight adiponectin, which is considered a more sensitive marker of metabolic dysfunction [20,43]. In the present study, no significant differences were observed; however, inhibitory tendencies were observed in liver weight (*p* = 0.07) and hepatic TG content (*p* = 0.06) after BCG vaccination (Table 1). Therefore, these results suggest that pre-vaccination with BCG not only delays the progression of glucose dysmetabolism, but also has beneficial effects on various diseases associated with lipid metabolism abnormalities, such as metabolic syndrome, in HFD-fed mice. 

Although innate immune cells are usually considered to be able to respond de novo to stimuli but not to form immunological memories, which have previously been considered only a part of adaptive immunity, it has been found that even organisms without an adaptive immune response can protect themselves against reinfection with pathogens [44]. “Trained immunity”, a term first coined in 2011, refers to the immunological memory responses of innate immune cells in response to past pathogen infections [45]. This phenomenon was clarified in humans, and it was found that BCG vaccination can lead to the epigenetic reprograming of monocytes, resulting in enhanced proinflammatory responses to secondary invasion with unrelated pathogens [46]. It has also been reported that BCG vaccination induces persistent epigenetic, transcriptional, and functional changes in hematopoietic stem and progenitor cells in human bone marrow [47]. Several studies have suggested that BCG-induced trained immunity may be partly related to the heterologous beneficial off-target effects of BCG vaccination [48,49,50]. Therefore, we assessed whether BCG-induced trained immunity modulates the progression of glucose intolerance in HFD-fed mice. Bone marrow cells were harvested from donor B6-Ly5.1 mice vaccinated with BCG or administered with PBS three weeks previously and transplanted into busulfan-conditioned mice. The progression of HFD-induced glucose intolerance was inhibited in recipient B6-Ly5.2 mice with> 90% myeloid cells derived from BCG-vaccinated mice compared with those with myeloid cells not affected by BCG (Figure 5). These data suggest that the inhibitory effects of BCG vaccination on glucose intolerance in HFD-fed mice may be at least partially mediated by trained immunity. Faustman and colleagues reported that in vitro and in vivo BCG treatments can improve the baseline glucose transport of monocytes, which is deficient in T1DM individuals [51], and also reported that the glucose uptake of peripheral monocytes isolated from T2DM subjects and the bone marrow cells of T2DM models of *db/db* mice were augmented by in vitro BCG treatment [19]. Therefore, it is suggested that such changes in glucose metabolism in bone marrow cells and their derived myeloid cells are relevant to the results of this study. The main limitation of our study is that it did not identify the molecular mechanisms and key functional cells for the inhibitory effects of BCG vaccination on glucose metabolism abnormalities, and these remain to be addressed in future studies.

## 5. Conclusions

Our study aimed to investigate, for the first time, the effects of prior BCG vaccination on glucose intolerance progression induced by HFD intake in normal mice. BCG vaccination significantly delayed the progression of glucose intolerance and tended to inhibit hepatic lipid accumulation and reductions in the glucagon-to-insulin ratio, suggesting a beneficial effect of BCG on the development of T2DM and NAFLD, which are frequently associated with features of metabolic syndrome. Furthermore, it has been suggested that the effects of BCG vaccination may be at least partially due to immune memory and trained immunity for hematopoietic stem and progenitor cells of bone marrow. In this study, we used the BCG Tokyo 172 strain to determine effects on abnormalities in glucose metabolism. However, it has been suggested that the BCG dose, BCG strain, and timing of BCG administration are important for achieving efficacy in human T1DM [17]. Further studies are required to understand the effects of differences in dose administration, timing, and strains of the BCG vaccine, together with the identification of the molecular mechanisms and key functional cells of the beneficial effects of BCG vaccination.

## Figures and Tables

**Table 1:** Growth and serum parameters in chow- and high-fat-diet-fed mice at the end of the experimental period.

Parameters	Chow	Cntl	BCG
Growth			
Final body weight (g)	27.5 ± 0.6 ^a^	39.7 ± 1.8 ^b^	36.6 ± 1.5 ^b^
Liver weight (g)	0.95 ± 0.03 ^a^	1.70 ± 0.19 ^b^	1.30 ± 0.08 ^ab^
Serum			
Triglyceride (mg/dL)	54.4 ± 4.9 ^a^	69.7 ± 3.3 ^b^	60.3 ± 6.1 ^b^
Total cholesterol (mg/dL)	160 ± 12	188 ± 8.6	161 ± 9.5
AST (IU/L)	29.3 ± 5.2	26.9 ± 5.6	21.8 ± 5.9
ALT (IU/L)	4.41 ± 1.24	4.02 ± 0.50	4.47 ± 0.44
Hepatic			
Triglyceride (mg/g liver)	31.3 ± 0.6 ^a^	57.4 ± 6.6 ^b^	40.6 ± 5.6 ^b^

Chow: chow group; Cntl: control group; BCG: BCG group; AST: aspartate aminotransferase; ALT: alanine aminotransferase. Data are shown as the mean ± SEM. Different letters indicate significant differences among the experimental groups using Tukey–Kramer multiple comparison test (*p* < 0.05).

## Data Availability

The data presented in this study are available in this article.
